# PET Foils Functionalized with Reactive Copolymers as Adaptable Microvolume ELISA Spot Array Platforms for Multiplex Serological Analysis of SARS-CoV-2 Infections

**DOI:** 10.3390/s24237766

**Published:** 2024-12-04

**Authors:** Sylwia Pniewska, Marcin Drozd, Alessandro Mussida, Dario Brambilla, Marcella Chiari, Waldemar Rastawicki, Elżbieta Malinowska

**Affiliations:** 1 Department of Medical Diagnostics, Centre for Advanced Materials and Technologies CEZAMAT, Warsaw University of Technology, 02822 Warsaw, Poland; 2 Chair of Medical Biotechnology, Faculty of Chemistry, Warsaw University of Technology, 00664 Warsaw, Poland; 3 Institute of Chemical and Technological Science “Giulio Natta”, National Research Council of Italy, 20131 Milan, Italy; 4 Department of Bacteriology and Biocontamination, National Institute of Public Health NIH—National Research Institute, 00791 Warsaw, Poland

**Keywords:** microvolume ELISA, PET, flexible substrate, multiplexing, protein microarray, immunoassay, COVID-19, serological biomarkers, IgG, nucleoprotein

## Abstract

Microvolume ELISA platforms have become vital in diagnostics for their high-throughput capabilities and minimal sample requirements. High-quality substrates with advanced surface properties are essential for these applications. They enable both efficient biomolecule immobilization and antifouling properties, which are critical for assay sensitivity and specificity. This study presents PET-based microvolume ELISA spot arrays coated with amine- and DBCO-reactive copolymers MCP-2 and Copoly Azide. The platforms were designed for the sensitive and specific detection of specific antibodies such as COVID-19 biomarkers. Supporting robust attachment of the SARS-CoV-2 nucleoprotein (NP), these arrays outperform traditional approaches. It was demonstrated that covalent attachment methods proved more efficient than passive adsorption, together with the reduction of non-specific binding. Analytical performance was verified with classical ELISA and real-time Surface Plasmon Resonance (SPR) analysis. It enables sensitive detection of IgG and IgA antibodies, including IgG subclasses, in human serum. Clinically, the platform achieved 100.0% sensitivity and 92.9% specificity for anti-NP antibody detection in COVID-19-positive and negative samples. Additionally, DNA-directed immobilization extended the platform’s utility to multiplex serological measurements. These findings underscore the potential of PET-based microvolume ELISA arrays as scalable, high-throughput diagnostic tools suitable for detecting multiple biomarkers in a single assay and easily integrated into microfluidic devices.

## 1. Introduction

Biomarker detection is vital in medical diagnostics. It provides critical insights into physiological or pathological processes and responses to treatments. Traditional single-analyte assays like ELISA are highly sensitive. However, they are limited by relatively low throughput, large sample consumption, and the inability to simultaneously detect multiple biomarkers [1]. The growing demand for miniaturized and multiplex detection platforms addresses this limitation. They enable parallel analysis of various biomarkers, providing a more comprehensive disease profile while reducing reagent use and cost [2]. Recent advances in microarrays and microfluidics have revolutionized biomarker detection [3,4,5,6], allowing high-throughput, automated assays. However, challenges in ensuring reliability and sensitivity and managing issues like bioreceptor immobilization, signal interference, and background noise still need to be carefully considered.

Viral infections induce the production of various biomarkers, including viral genetic material, proteins, and host-derived antibodies across different isotypes and subclasses, which indicate an immune response [7]. The N and S proteins are primary targets of the humoral response [8], making them suitable as receptors for antibody detection in diagnostic platforms. Differentiating antibody classes and subclasses provides insight into infection progression and immune response [9]. The IgM antibody isotype is the first to respond, while IgG is the most abundant, with IgG1 and IgG3 crucial for viral defense [10]. In SARS-CoV-2 infection, IgM, IgA, and IgG are detectable soon after seroconversion [11,12].

To accurately reflect the complexity of biological processes, such as the progression of infection, systems capable of detecting multiple biomarkers in a single assay are essential. Established methods, such as lateral flow immunoassays [13,14], fluorescence-based colorimetric sensing [15,16], and label-free biosensors [17,18] are crucial in advancing multiplex biosensing. They enable rapid and sensitive detection across a range of applications. Research on SARS-CoV-2 antibody detection and distribution has been extensive due to its vital role in developing serological diagnostics [19,20,21]. However, developing multiplex analytical platforms presents distinct challenges. The effectiveness depends on accommodating a wide range of receptors, including recombinant proteins, antibodies, etc., and ensuring their proper binding with the assay substrate. Selecting an appropriate strategy for protein immobilization on solid supports is challenging due to the structural diversity of receptor proteins and the complexity of their interaction with surfaces [22]. For example, physical adsorption is simple but highly dependent on the protein’s structure, charge, and hydrophilicity, making it less reliable and prone to probe loss over time [23]. Covalent immobilization offers stronger attachment but requires more complex chemistries [24]. Affinity-based methods, like His-Tag [25] or Avidin-Biotin [26], ensure oriented attachment but are expensive and complicated. As a result, ongoing efforts focus on developing efficient one-step protein coupling methods that also prevent nonspecific adsorption. In turn, the ideal substrate for multiplex assays should support stable, specific immobilization of diverse bioreceptors while preserving their activity. Such a substrate has reactive groups for easy conjugation and antifouling properties, ensuring high sensitivity and specificity. Compatibility with various detection methods, such as electrochemical and optical, is also essential for high-throughput point-of-care treatment (POCT) devices.

A promising solution is the development of microvolume assays on transparent polyester substrates, known as lab-on-a-foil platforms. This miniaturized, adaptable format offers several advantages, including cost-effectiveness through reduced reagent use and seamless integration with microfluidic channels and microarrays on the same substrate. It enables facile sample processing and analysis. Adapting PET-based arrays into microfluidic cassettes is relatively straightforward. It allows for the fabrication of sample-to-answer platforms [27]. The transparency of polyester foils facilitates the usage of optical detection methods such as fluorescence and colorimetry. Lab-on-a-foil devices are portable, making them ideal for POCT applications. PET foils can be coated with reactive copolymers to provide functional surfaces dedicated to bioreceptor immobilization, enhancing their application in multiplex assays. Such polymers and copolymers are typically equipped with reactive groups, like azides, amines, or quinones, to enable efficient “click” chemistry for stable, oriented biomolecule attachment [28,29]. Incorporating antifouling elements into the copolymer coating further enhances surface quality by reducing nonspecific binding from complex biological samples. This improvement increases assay sensitivity and specificity by minimizing background interference.

This work explored the feasibility of using reactive copolymers, MCP-2 and Copoly Azide, to coat PET substrates for developing flexible serological microvolume ELISA spot arrays. Two immobilization strategies (e.g., covalent amine coupling and DNA-directed immobilization (DDI)) were applied to immobilize receptor proteins, with conventional passive adsorption as a reference. The PET-based platforms were assessed for their ability to detect different immunoglobulin classes and subclasses in sera from SARS-CoV-2 patients. The developed solution offers an effective distinction between positive and negative samples. The clinical potential of these immunoassays was also evaluated. Furthermore, immunoassays based on DDI for multiplex detection of viral biomarkers were designed, demonstrating the adaptability of the platform. Methods such as ELISA and Surface Plasmon Resonance (SPR) were employed to investigate the mechanisms of layer formation and immunoreaction pathways. We believe that the designed platforms possess high potential for further development and application in biosensing and microfluidics-integrated diagnostics.

## 2. Materials and Methods

A detailed list of basic chemical and biochemical reagents, as well as detailed oligonucleotide sequences used within this study, can be found in the Supplementary Materials.

MCP-2 and Copoly Azide copolymers were synthesized as reported elsewhere [30]. Transparent poly(ethylene terephthalate) foils for laser printers (Diamax, A4, d = 100 µm) were sourced from Argo (Warsaw, Poland). ARcare^®^ 90445Q transparent, flexible adhesive tape was sourced from Adhesives Research Inc. (Glen Rock, PA, USA). Gold SPR slides (50 nm gold layer thickness) were purchased from Horiba Scientific (Palaiseau, France). DNA oligonucleotide sequences were purchased from Metabion GmbH (Planegg, Germany). All solutions were prepared using deionized (DI) water (conductivity < 0.055 μS/cm at 20 °C, TOC < 1.0 ppb). Human serum samples were obtained through a scientific collaboration with the National Institute of Public Health NIH—National Research Institute (Warsaw, Poland). “Positive” sera were collected from individuals infected at the time with the SARS-CoV-2 virus or had either recovered from COVID-19, while “negative” sera were sourced from healthy individuals before the outbreak of the pandemic. The Bioethics Committee of NIPH-NIH approved the study. Participants were informed about the study and gave consent to use the sample. Reference analyses of serum samples, classifying them as positive or negative, were conducted using standard serological ELISA and RT-PCR tests. Detailed protocols for conjugating receptor proteins with ssDNA anchors, purification of the conjugates, and procedures for classic ELISA assays and SPRi studies are provided in the Supplementary Materials.

### 2.1. Fabrication of Polymer-Coated PET Foils

Prior to MCP-2/Copoly Azide polymer coating, hydrophilization of the bare PET foil surface was performed using a 10 min treatment with UV/ozone cleaner (Ossila, Sheffield, UK). Copoly (DMA-NAS-MAPS), whose commercial name is MCP-2, is a copolymer obtained by free radical polymerization of *N,N*-dimethylacrylamide (DMA, 97% molar fraction), N-acryloyloxysuccinimide (NAS, 2% molar fraction), and 3-(trimethoxysilyl)propyl methacrylate (MAPS, 1% molar fraction). The principal constituent of Copoly (DMA-NAS-MAPS), dimethylacrylamide (DMA), forms the polymer backbone and ensures strong passive adsorption to variety of materials. NAS is the chemically reactive monomer responsible for probe immobilization through nucleophilic substitution with amine groups on biomolecules. The same monomer is exploited in post-polymerization modifications. In these reactions, NAS is reacted with linkers that contain amine groups to introduce novel functionalities into the polymer backbone. Using this strategy, Copoly Azide was synthesized. Specifically, after the polymerization of DMA, NAS, and MAPS (95, 4, and 1% molar fraction, respectively) the obtained polymer was reacted with 2.5 equivalents of 11-azido-3,6,9-trioxaundecan-1-amine, as described elsewhere. The MCP-2 and Copoly Azide structures are shown in Figure S1. The layer obtained with reactive copolymers has a dual role: antifouling properties due to blocking surface sites via hydrogen bonds, and bearing reactive groups that enable controlled interactions with biomolecules. The functional groups of the copolymers were characterized using FT-IR and NMR analysis [30].

For coatings, copolymers were dissolved in DI water to a final concentration of 2% *w*/*v* and then diluted 1:1 with a solution of 1.6 M ammonium sulfate. The MCP-2 polymer was used for the direct covalent immobilization of receptor proteins. The Copoly Azide polymer is capable of click-chemistry reactions with DBCO-conjugated DNA probes. This enables DNA-directed immobilization of protein–oligonucleotide conjugates. The foil was dip-coated for 1 h with the chosen copolymer, then rinsed with DI water, dried under compressed air, and cured under vacuum at 80 °C for 15 min. The immobilization in predefined spots was possible thanks to the adhesive tape used to produce the rims of microwells. Holes, 5 mm in diameter, positioned in 96-well plate format, were cut with a VLS2.30 CO_2_ laser plotter (Universal Laser Systems, Vienna, Austria).

The coating of PET foil coating with MCP-2 and Copoly Azide involves the passive attachment of the copolymers through weak, non-covalent interactions such as hydrogen bonding, Van der Waals, and hydrophobic interactions with the surface. Following dip coating, polymerization occurs, forming a stable and robust polymer layer that enables further immobilization of biomolecules. The morphological and chemical properties of passive coating have already been examined by means of atomic force microscopy and X-ray photoemission spectroscopy [31].

### 2.2. Protein Immobilization on PET

Protein receptor immobilization on modified PET substrates was achieved in two ways: through direct coupling to amine-reactive MCP-2 and indirectly via DNA-directed immobilization using oligonucleotide probes tethered to Copoly Azide [30]. For covalent attachment of proteins on MCP-2-coated foil, a 20 µg/mL solution of SARS-CoV Nucleocapsid recombinant protein (or another SARS-CoV-2 antigen) in PBS was dispensed onto microarray spots (11 µL each) and incubated overnight at 4 °C in a humid atmosphere. The remaining reactive sites were then blocked by applying 20 µL of PBST with 3% BSA for 30 min, followed by rinsing with PBST and drying under compressed air. For DDI on Copoly Azide-coated foil, a 20 µg/mL solution of DBCO-modified DNA in DI was dispensed onto microarray spots (11 µL each) and incubated overnight at 4 °C in a humid atmosphere. Chromatographically purified fractions of ssDNA conjugates with recombinant SARS-CoV-2 nucleoprotein or monoclonal anti-human IgG antibody (~150 nM in 50 mM phosphate buffer, pH 7.2, with 150 mM NaCl) were then dispensed as 11 µL spots onto the DNA-coated foil. After 1 h of incubation, the substrate was rinsed with PBS containing 10 mM Mg(NO_3_)_2_ and dried under compressed air. The blocking steps were identical to those used for covalent immobilization.

### 2.3. ELISA-like Assays on PET Foils

Two types of immunoreactions were performed in the prepared microwells on PET foils. Indirect immunolabeling was used to detect immunoglobulin subclasses in human sera, utilizing specific monoclonal mouse antibodies and an anti-mouse IgG–alkaline phosphatase conjugate. In contrast, direct immunolabeling with an anti-human IgG/IgA–alkaline phosphatase (ALP) conjugate was employed to detect total IgG and IgG/IgA classes of anti-NP antibodies in human sera. The detailed composition of each assay is provided in Section 3. According to the general protocol, 11 µL of the diluted serum or appropriate target antibody was incubated for 1 h at room temperature in a humid atmosphere, rinsed with PBST, and dried under compressed air. Then, 11 µL of the antibody–ALP conjugate (direct assay) or appropriate secondary antibodies (indirect assay) in PBST buffer with 3% BSA were added to each well, incubated for 1 h at room temperature in a humid atmosphere, rinsed with PBST, and dried under compressed air. Proper antibodies were used at 1 µg/mL, and the antibody–ALP conjugate at 3 µg/mL. For the indirect assay, the antibody–ALP conjugate was added in the third step (incubation and dilution procedure the same as for the direct assay). For quantitative analysis, 10 mM freshly prepared *p*-nitrophenyl phosphate (PNNP) in 50 mM carbonate buffer pH 9.6 was used as the ALP substrate. An enzymatic reaction was initiated by dispensing 11 µL of PNNP substrate into each microwell, followed by incubation in a humid atmosphere for 12 min. Subsequently, 8 µL of substrate solution was collected from each spot and transferred to a multi-well plate containing 150 µL of 50 mM carbonate buffer (pH 9.6). Absorbance was measured at 405 nm using a Multiskan Go microplate reader (Thermo Scientific, Waltham, MA, USA).

## 3. Results and Discussion

To achieve the maximum recognition capacity of immunosensors and guarantee the long-term stability of the receptor proteins on the substrate, efficient immobilization in a well-defined orientation is of great value. However, immobilizing protein receptors for rapid diagnostic platforms remains a complex challenge. Antibodies, due to their larger size and structural complexity, are generally easier to immobilize. Their multiple functional groups, such as amines, carboxyl groups, and hydrophobic domains, enable stable and oriented surface attachment [32]. Antibodies also exhibit greater stability and robustness under various conditions compared to antigens. Their structural and functional similarity ensures a consistent, repeatable immobilization. This uniformity allows for standardized protocols to be applied universally and supports reliable and reproducible immobilization across different immunoplatforms [33]. In contrast, the structural and physicochemical diversity of antigens makes developing immobilization protocols more challenging.

**Figure 1:**
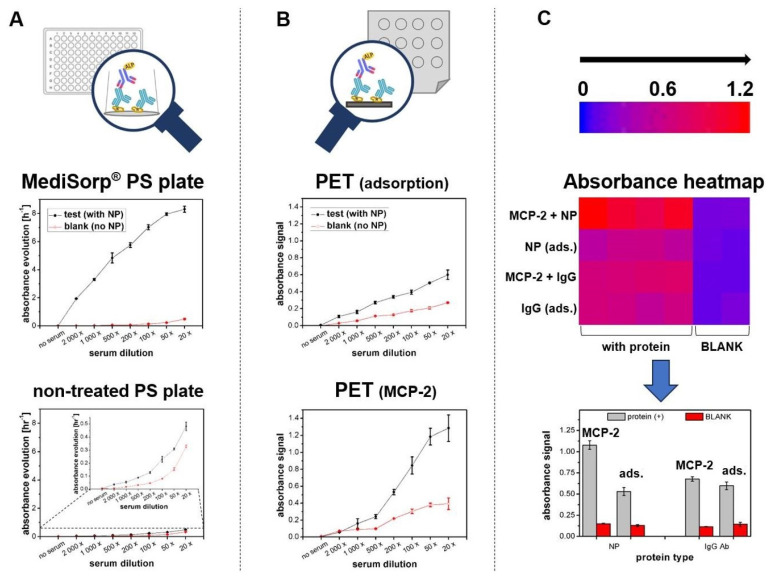
The effect of the substrate type ((**A**)—polystyrene, (**B**,**C**)—PET foils) on NP immobilization efficiency and non-specific adsorption of human serum. The red points (**A**,**B**) represent immunoassay responses obtained for the control wells (without the immobilized receptor protein), and the black points correspond to the total responses obtained for the test wells. Heat map (**C**) represents raw absorbance signals obtained for assays performed with two immobilized receptor proteins (two first rows—NP, two bottom rows—anti-human IgG). The first row for each protein represents covalent immobilization on MCP-2, and the second corresponds to passive adsorption. The layout on the plate is shown as 4 test wells + 2 wells of BLANK (without immobilized receptor protein). Mean signal values were summarized as a bar graph in (**C**).

To illustrate the impact of substrate type on serological immunoassay sensitivity and non-specific adsorption of serum components, response profiles for assays on standard multi-well plates (Figure 1A) and PET-based miniaturized substrates (Figure 1B) were compared. The efficiency of passive immobilization of nucleoprotein (NP) was evaluated using a classical untreated polystyrene plate and a commercial MediSorp^®^ plate designed for protein binding. Additionally, the contribution of non-specific binding of serum proteins to the ELISA signal was assessed. It can be seen in Figure 1A that the MediSorp^®^-modified plate exhibited approximately 16 times higher assay sensitivity with minimal non-specific binding compared to the untreated polystyrene plate. The unmodified polystyrene showed low antigen coating capacity and high non-specific protein adsorption, with non-specific interactions contributing up to 70% of the total response at higher serum concentrations. These results highlight the strong dependence of passive adsorption efficiency on surface properties, especially its hydrophilicity [34].

The results obtained for the MediSorp^®^ plates are widely regarded as the gold standard for immunoplatform design. However, adapting this type of substrate to other applications, such as microfluidics, is challenging. To address this limitation, the use of PET foil as a flexible substrate for protein immobilization was investigated. Flexible substrates provide more versatility for incorporating immunoplatforms into complex diagnostic devices. For this purpose, two immobilization methods on PET foil were tested: passive adsorption and covalent attachment. Covalent binding was achieved by coating the foil with the amine-reactive copolymer MCP-2, which enables stable protein attachment. Both MCP-2 and its N_3_-functionalized derivative Copoly Azide are distinguished by ease of coating on solid surfaces without requiring an activation step.

As shown in Figure 1B, using the amine-reactive MCP-2 polymer improved protein immobilization efficiency over twofold compared to passive adsorption. Additionally, non-specific binding remained low and stable across higher serum dilutions. At the highest serum concentration, non-specific signals accounted for only 30.1% of the total response, compared to 46.5% for passive adsorption, demonstrating the effective antifouling properties of the MCP-2 layer against serum components.

The effectiveness of the MCP-2 polymer for covalent immobilization is especially evident with recombinant protein attachment. As illustrated by the heat map and graph in Figure 1C, covalent immobilization of NP on the MCP-2 polymer shows approximately 50% higher efficiency (measured as increased immunoassay signal) compared to passive adsorption on unmodified PET, with similar levels of non-specific adsorption. This effect is less pronounced for antibody immobilization, with a difference of under 9% in favor of MCP-2. This is presumably due to the large contribution of hydrophobic interactions affecting the efficient adsorption of immunoglobulins on PET and other hydrophobic materials [35].

The results indicate that covalent immobilization via an amine-reactive polymer is particularly well suited for the effective immobilization of antigens in developing serological assays. The practical advantages of coating with reactive copolymers over traditional surface modifications like APTES silanization are also significant. Copolymer coatings provide greater flexibility in modifying surface properties and enable more controlled and uniform layers. Additionally, the single-step process is easier to scale up and automate, ensuring better consistency and reproducibility. In contrast, widely employed ω-functionalized silanes require a complex two-step process (coating and glutaraldehyde activation) with strict humidity and reaction time requirements. It makes them labor-intensive and challenging to scale up for microfluidic and other technological applications [36].

Simultaneous detection of multiple viral biomarkers is essential for understanding infection dynamics. Inflammation triggers complex immune responses, with viral proteins and host antibodies appearing at different stages. Multiplex assays facilitate the identification of viral antigens and a broad range of antibody classes and subclasses, providing insights into viral load and immune activity. This comprehensive approach enables more accurate diagnostics, differentiation between acute and past infections, and improved monitoring of disease progression or treatment efficacy. Therefore, flexible serological platforms should support detecting various antibodies against diverse antigens and employ multiple assay formats. To this end, MCP-2-coated substrates were thoroughly evaluated for their suitability in diverse serological immunoassays. The experiments began with selecting a suitable model antigen (Figure S2). Results showed that platforms using NP and RBD proteins as receptors offer comparable serum antibody detection sensitivity, with RBD showing only a 3% lower response than NP for the same sample. In contrast, the S1 antigen exhibited nearly 48% lower sensitivity compared to NP and a noticeably higher non-specific signal. These findings confirm the compatibility of MCP-2 with various recombinant antigens. The lack of response for the S1/S2 protein-based assay was attributed to the poor antibody–antigen affinity, as confirmed by a reference test on the MediSorp^®^ polystyrene substrate.

**Figure 2:**
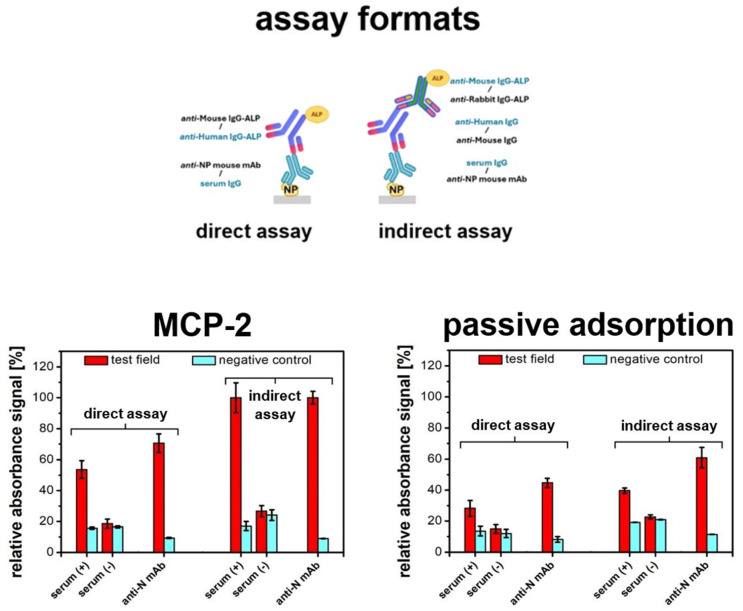
Comparison of direct vs. indirect assays for two protein immobilization methods on PET foil: covalent (MCP-2) and passive adsorption. Assays were conducted using human samples (SARS-CoV-2-positive and -negative sera) and anti-SARS-CoV-2 NP monoclonal antibodies. Red bars show the results of test wells (“positive” serum or monoclonal antibody solution), and blue bars represent control wells (negative serum or buffer). Values are expressed as normalized absorbance signals. Black labels in the graphical schematic refer to serum IgG assays, while blue labels indicate the detection of monoclonal anti-NP IgG.

NP was selected as the model antigen to detect specific antibodies. The experiment shown in Figure 2 compared immobilization efficiency via MCP-2 vs. passive adsorption for two assay formats: direct and indirect. The choice of format depends on the availability of specific antibody conjugates. Indirect assays are generally more versatile and sensitive due to additional signal amplification during the secondary labeling but require an additional step, which complicates the entire process. We anticipate that the developed PET-based platforms will support versatile detection, regardless of the chosen assay format. The expectations described above were validated by assay results for both immobilization methods—passive and covalent attachment. A similar trend was observed across both formats: the highest signals were detected for monoclonal anti-NP IgG compared to human serum anti-NP IgG, with the indirect assay format providing over 30% greater sensitivity than the direct format. Non-specific adsorption remained low for both methods. These findings confirm the superiority of covalent NP immobilization using MCP-2 over passive adsorption across various serological immunoassay formats. Covalent immobilization provides robust attachment of receptor proteins. However, it usually requires complex surface modification that poses technological difficulties. Passive adsorption, while simple, often shows immobilization efficiency limitations [37]. PET foils are compatible with various detection strategies, but maintaining their structural integrity may be challenging. Already described methods of their chemical modification usually require harsh conditions such as high temperatures [38], involve multiple reagents [39,40,41], and are time- and labor-consuming [42,43]. Coating with reactive copolymer MCP-2 is a simple procedure that occurs under mild conditions and allows for one-step covalent attachment of receptor proteins while preserving the good mechanical properties of the PET substrate.

In serological assays, the complexity of biological samples poses significant challenges due to the potential for non-specific adsorption of proteins and other biomolecules to the surface of diagnostic platforms. This non-specific binding can lead to elevated background signals, reduced assay sensitivity, and compromised specificity, resulting in false positive results, ultimately affecting the reliability and accuracy of diagnostic results [44,45,46]. Studies on possible blocking agents in immunoassays showed polymeric compounds’ superiority in reducing background noise [47]. *N,N*-dimethylacrylamide-derived copolymers such as MCP-2 have been used in microarrays and biosensors, demonstrating rapid surface adhesion, effective covalent biomolecule attachment, and antifouling properties [48]. Compared to traditional surface functionalization methods, covalent attachment to polymer-coated surfaces enhances binding efficiency and reduces non-specific interactions [49].

**Figure 3:**
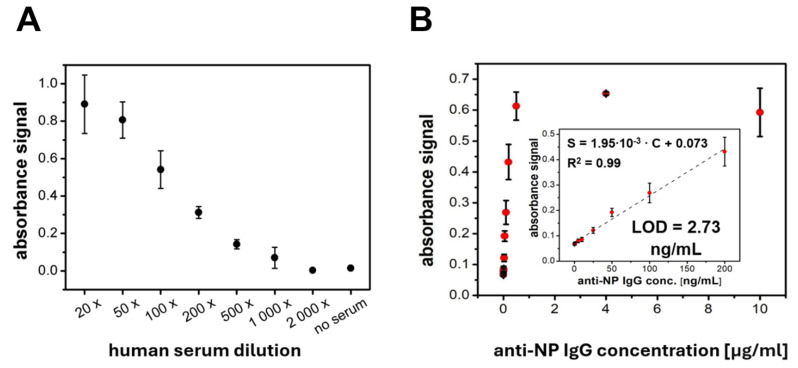
The plot of immunoassay response vs. dilutions of COVID-19-positive human serum (**A**) and concentration of mouse monoclonal anti-NP antibodies (**B**). The inset provides a magnified view highlighting the linear response range and key analytical parameters.

Further studies have validated the use of the MCP-2-based PET immunoplatform for quantitative analysis. As shown in Figure 3, covalently immobilized NP proteins on polymer-coated substrates enabled quantitative detection of both human anti-SARS-CoV-2 NP antibodies in serum (Figure 3A) and mouse monoclonal anti-NP antibodies (Figure 3B). The response profile to increasing serum dilutions showed a dynamic range of 20× to 1000×. It covers typical working dilutions used in routine serological ELISAs [50,51]. Based on the calibration curve shown in Figure 3B, the analytical parameters of the direct immunoassay for anti-NP monoclonal antibodies as a model target were evaluated. Linearity of the concentration-response curve was observed within the range of 5–200 ng/mL, with a lower detection limit of 2.73 ng/mL. The presented analytical characteristics of MCP-2-based immunoplatforms confirm their suitability for serological quantitative analysis. However, as with classical ELISA formats, the assay’s sensitivity can be adjusted at the expense of analysis time, for example, by modifying incubation durations or enzymatic reaction times (currently set to 12 min).

**Figure 4:**
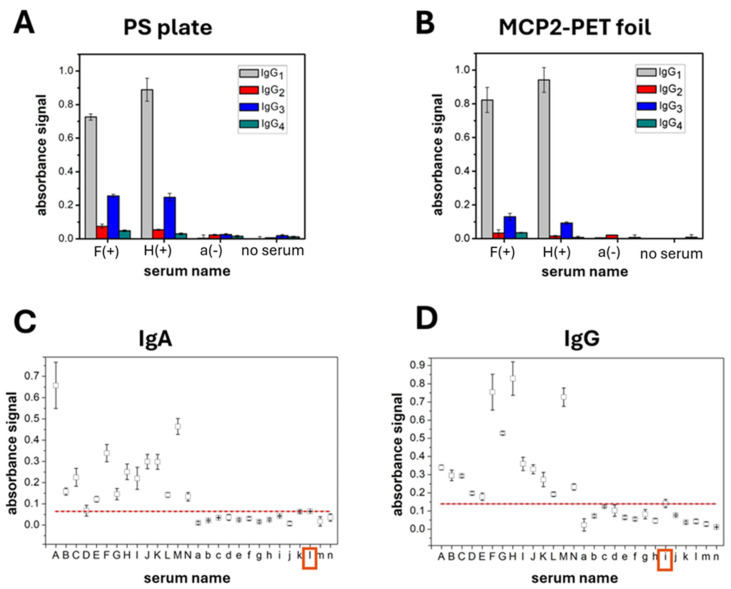
Analysis of antibody subclasses in human sera using standard ELISA (**A**) and PET foil-based ELISA with MCP-2 coating (**B**). The bar graph shows absorbance signals for IgG1, IgG2, IgG3, and IgG4 subclasses in positive, negative, and control samples using an indirect assay. Detection of antibody classes: IgA (**C**) and IgG (**D**) against SARS-CoV-2 NP in human sera. Boxplots show absorbance signals for various sera samples, with uppercase letters representing “positive” sera and lowercase letters representing “negative” sera. The red dashed line indicates the cutoff value differentiating positive and negative samples.

The occurrence of specific IgG subclasses (IgG1, IgG2, IgG3, and IgG4) in human sera was examined using an indirect assay. SARS-CoV-2 NP was immobilized on two substrates: the ELISA-dedicated MediSorp^®^ plate (Figure 4A) and PET foil coated with MCP-2 (Figure 4B). These platforms were used to capture and identify subclass-specific IgG responses. The indirect format allows differentiation of each IgG subclass. It provides not only confirmation of antibody presence but also detailed insights into their specific subclass distribution.

Two randomly selected COVID-19-positive sera (named F and H) with recent infection and well-characterized antibody profiles were analyzed. The results showed a dominant presence of IgG1, significantly lower levels of IgG3, and minimal amounts of IgG2 and IgG4 (in one serum sample IgG2 and IgG4 were barely detectable). The COVID-19-negative serum (named a) and blank (no serum) both exhibit minimal absorbance across all IgG subclasses, which confirms the specificity of the assay. These findings align with the typical distribution of IgG subclasses in human serum, where IgG1 accounts for approximately 60–70% of the total IgG [52]. IgG1 and IgG3 are the main subclasses produced in response to viral infections, while IgG2 and IgG4 are less abundant. IgG2 typically responds to polysaccharide antigens linked to bacterial infections. The dominance of IgG1 and, to a lesser extent, IgG3 in the assay aligns with the expected immune response to viral infections. Differences in subclass profiles among positive sera reflect variability in individual immune responses. While both positive sera show a strong presence of IgG1, the relative levels of IgG3, IgG2, and IgG4 differ between the two samples. However, the trend in the distribution of individual subclasses is noticeable regardless of the type of immunoassay (PS plate vs. flexible substrate). This variability can be attributed to factors such as the timing of sample collection, individual differences in immune system function, or varying levels of exposure to the virus [53,54]. The distinct profiles of these IgG subclasses underscore the importance of personalized immunological assessments.

To validate the diagnostic performance, the immunoplatform was used to test a large pool of serum samples from COVID-19-positive and -negative patients. Two antibody classes—IgA (Figure 4C) and IgG (Figure 4D)—with proven diagnostic value were selected. Analyzing both provides insight into the immune response and infection stage: IgG indicates a long-term, mature response, while IgA is key for mucosal immunity [55]. The platform differentiates between positive and negative samples using a cutoff value defined by Equation (1) [56].

Cutoff = Mean of Negative Samples + (2 × SD)(1)

The boxplots in Figure 4 show that absorbance signals for positive sera are significantly higher than those for negative samples, exceeding the cutoff value. This confirms that the MCP-2-based immunoplatform effectively evaluates IgG and IgA antibody levels against NP in serum samples. Variability in signal intensity among COVID-19-positive sera reflects differences in antibody concentrations between patients. The IgG/IgA ratio also varies, indicating different infection stages at the time of biological material collection [57]. COVID-19-negative sera generally showed signals below the cutoff, indicating low or absent IgA/IgG antibody levels compared to positive samples. Sensitivity, specificity, positive predictive value (PPV), negative predictive value (NPV), and assay accuracy are summarized in Table 1. MCP-2-based immunoassays demonstrated identical diagnostic parameters for both IgA and IgG classes. All 14 COVID-19-positive samples were correctly identified, yielding 100% sensitivity. For the 14 COVID-19-negative samples, one false positive was recorded (IgA sample “l” and IgG sample “i”), resulting in 92.9% specificity. These results suggest that the developed platforms are highly suitable for the immunodetection of SARS-CoV-2 serological biomarkers.

Although covalent immobilization is generally more efficient than passive adsorption, it does not fully address the challenges arising from antigen structural diversity. Both methods rely on direct interactions between protein functional groups and the surface, making successful binding highly dependent on protein structure and the availability of reactive groups. In contrast, DNA-directed immobilization (DDI) overcomes these limitations by using the hybridization of complementary DNA strands. It enables stable and straightforward immobilization of receptor proteins conjugated with single-stranded DNA onto pre-immobilized complementary ssDNA probes. The DDI strategy demonstrates significant potential for the precise construction of protein arrays with high selectivity and spatial resolution. Two effective approaches can be employed: one using DNA-conjugated receptor proteins with distinct DNA anchors for simultaneous immobilization through specific hybridization, and the other utilizing a unified DNA anchor with controlled spotting for spatial alignment. The first approach ensures selective and simultaneous immobilization of multiple proteins in a single flow step, while the second provides precise spatial control by directly depositing conjugates onto the surface. Both methods effectively prevent cross-interference during immobilization, guaranteeing high specificity and reproducibility. These solutions are particularly valuable for high-throughput biosensor array fabrication, facilitating advanced multiplex detection applications.

**Figure 5:**
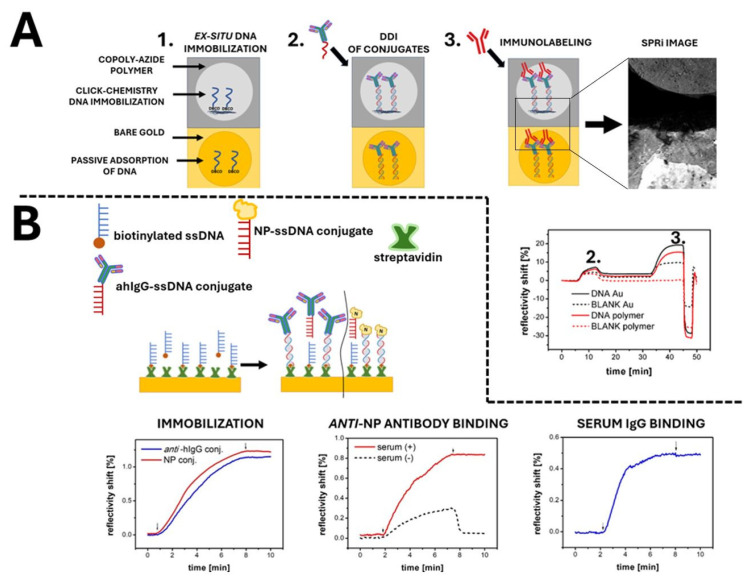
(**A**) Schematic and SPRi analysis of DNA-directed immobilization of antibody–DNA conjugates on Copoly Azide polymer. The upper half of the slide was coated with polymer, while the lower half remained uncoated as a reference (1). The SPRi sensogram shows real-time reflectivity shifts for antibody–DNA conjugate immobilization (2), immunolabeling with secondary antibodies (3), and layer regeneration. (**B**) SPRi responses for DNA-directed immobilization of nucleoprotein (NP) and anti-human IgG conjugates. The left graph compares anti-human IgG (blue) and NP (red) conjugate immobilization. The middle graph shows human anti-NP antibody binding from COVID-19-positive (red) and -negative (black dashed) serum. The right graph depicts human IgG binding from a COVID-19-negative serum.

The initial step of our studies involved comparing the efficiency of one-step covalent immobilization of ssDNA probes on reactive Copoly Azide polymer to passive adsorption using Surface Plasmon Resonance imaging (SPRi). For this purpose, half of the SPR slide was coated with Copoly Azide. The other half of the slide was left uncoated, and modified DNA probes were passively adsorbed onto bare gold. A schematic representation of the SPRi chip topography, along with a CCD image of the actual SPRi slide, is shown in Figure 5A.

SPRi measurements enable real-time monitoring of the immobilization of protein conjugates and their subsequent immunolabeling with antibodies. Additionally, SPRi facilitates the visualization of differential images, which reveal non-specific protein adsorption on the unmodified gold surface. The Copoly Azide polymer, visible on the upper part of the chip, demonstrates excellent antifouling properties and supports efficient covalent attachment of DNA probes for subsequent DNA-directed immobilization of protein conjugates. This is confirmed by the absence of brightening in the differential image and no change in reflectance for the polymer-coated area without immobilized DNA (red dashed line). Brightening is observed only at the specific spot where ssDNA was pre-immobilized (solid red line), indicating high specificity of receptor binding. In the case of bare gold, the brightening of the differential image and the significant change in reflectance (black dashed line) are significantly due to the non-specific adsorption of antibodies to the uncoated surface. The critical advantage of the DDI-based platforms is the possibility of rapid and effective layer regeneration which allows for its renewed usage [58]. As can be seen in the final section of the sensogram, DDI-based immunocomplexes on Copoly Azide can be quickly and efficiently removed from the surface by the injection of 50 mM NaOH. After regeneration, the DNA probes can be successfully reused to construct subsequent receptor layers via DDI.

In the next step, DDI was used to immobilize two DNA–protein conjugates (NP and anti-human IgG antibody) onto the surface of a gold SPR transducer (Figure 5B). The immobilization sensograms showed distinct responses corresponding to the surface density of the immobilized proteins. Notably, protein loading was similar for both conjugates and could be regulated by adjusting the density of DNA probes on the transducer surface [59]. The anti-human IgG antibody conjugate in the receptor layer allowed the capture of various IgG antibodies from COVID-19-negative human serum. Stable binding of the serum IgG antibody can be seen in the sensogram, with no apparent dissociation. In turn, immobilized NP conjugate effectively captured specific antibodies from the COVID-19-positive serum. No binding was observed with the negative serum, indicating high specificity of the DDI-based serological assay. The negative serum exhibited only a reversible, non-specific interaction during the association stage, with immediate dissociation upon returning to the running buffer. This confirms that the surface layer is not entirely resistant to biofouling; however, this does not negatively affect its potential application in serological biosensing. In contrast, the positive serum showed a stable increase in reflectivity. This confirms antibody capture by the immobilized antigen. DDI enabled rapid, stable immobilization of both protein receptors and differentiation of actual samples.

The use of SPR allowed for comprehensive testing of all components involved in DDI, providing real-time monitoring of the assembly process. This approach enabled the tracking of each step and supported the transition from substrates designed for label-free detection to flexible PET foils adapted for label-based detection. As shown in Figure 1C, the reactive MCP-2 copolymer enables easy and efficient covalent immobilization of recombinant proteins, although the efficiency for antibodies was moderate. The DDI approach is expected to eliminate dependence on receptor protein structure during immobilization.

**Figure 6:**
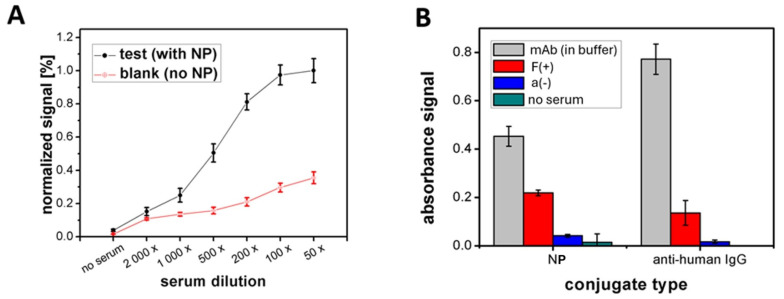
Serological assay results using DNA-directed immobilization on PET foil coated with Copoly Azide. (**A**) Normalized absorbance as a function of serum dilution for anti-NP antibody detection. The black line shows test wells with the NP conjugate, while the red line represents control wells without receptor proteins. (**B**) Multiplex assay showing absorbance signals for anti-NP antibodies and human IgG from various sources, including monoclonal antibodies, COVID-19-positive serum, and COVID-19-negative serum.

To assess the potential of PET foil coated with Copoly Azide for constructing flexible, easily adjustable immunoassays via DDI, the NP–DNA conjugate was immobilized on the coated substrate. DBCO-modified DNA on the polymer-coated foil enabled hybridization with complementary strands, allowing rapid attachment of the DNA-labeled receptor protein. A quantitative serological assay was then conducted using human serum at various dilutions (Figure 6A). As can be observed, the signal increases with decreasing serum dilution. It indicates a higher concentration of antibodies in the serum, thus confirming the feasibility of the receptor layer obtained by means of DDI. No significant non-specific binding occurred for controls without receptor proteins on the surface. The obtained blank signals (Figure 6A, red line) remained low and showed only a slight increase as the serum protein concentration increased, confirming the high specificity of the assay. A detailed study of the specificity of immunoplatform formation via DDI (Figure S3) showed that protein receptor immobilization on Copoly Azide occurs almost exclusively through DNA–DNA duplex formation. Non-specific adsorption contributes less than 10% to the total signal in all tested cases. The antifouling properties of Copoly Azide are satisfactory, which supports the construction of DDI-based platforms for sensitive and specific serological assays. Most importantly, these platforms enable the development of multiplex assays using various protein-based receptors immobilized through a straightforward single-step process. This was demonstrated by the simultaneous immobilization of two distinct protein conjugates (NP as a recombinant antigen and anti-human IgG as an antibody representative) within a single immunoplatform (Figure 6B). The developed immunoassays were used to detect specific anti-NP antibodies and total IgG antibodies in two human serum samples (COVID-19-positive and -negative). The designed assay allows distinguishing between samples and confirms the absence of non-specific binding. The demonstrated potential of multiplexing is crucial for developing more comprehensive diagnostic assays to detect multiple biomarkers in a single test.

To understand the strengths and limitations of antigen immobilization in micro-spot ELISA, analyzing their mechanisms and challenges is essential. Covalent immobilization on MCP-2-coated PET involves the reaction between amine groups of the protein and NAS groups in the copolymer, forming stable amide bonds that ensure robust attachment and minimize protein loss during washing. Despite its simplicity, this method can partially affect sensitive proteins, and attachment efficiency depends on receptor structure [60]. Passive adsorption relies on non-covalent π–π hydrophobic interactions, making it easy to implement but variable due to protein and surface properties [61]. Proteins bound this way may desorb or rearrange, impacting assay reliability. DDI uses affinity-based attachment by hybridizing complementary probes, providing precise orientation and multiplexing capability. However, DNA duplex stability is affected by ionic strength and temperature, making washing with low-ionic solutions problematic and requiring consistent conditions to maintain attachment integrity [62].

The potential of the developed platform extends beyond SARS-CoV-2 detection and holds promise for broader use in multiplex diagnostics. Its adaptability for integrating various protein bioreceptors suggests that this platform could be tailored to determine different biomarkers rapidly, supporting POCT where timely and accurate results are critical. The robust binding properties and minimal non-specific interactions make it an attractive tool for multi-analyte screening. PET foil-based immunoplatforms can be easily integrated with microfluidic systems to further develop high throughput diagnostic tools [63,64]. The inherent properties of these flexible substrates, such as ease of cutting and reshaping, and wide working temperature and pressure range, make them highly adaptable for such applications [65]. The platform can be transformed into a fully functional microfluidic device by incorporating laser-cut microchannels and bonding methods such as thermal lamination or adhesive tapes. These techniques allow the fabrication of sealed cassettes with embedded microchannels, facilitating controlled reagent flow and supporting automated sample processing [27]. The possibility of integration and the compatibility with common optical detection methods leverage potential of the developed platform for the construction of multiplexed diagnostic assays. Exploring these capabilities in further studies should demonstrate the versatility of the immunoplatforms and enhance scalable and sensitive immunodiagnostics.

## 4. Conclusions

In this work, we have systematically studied the impact of receptor protein characteristics on immobilization strategies and evaluated the suitability of reactive copolymer-coated PET as a substrate for constructing flexible, microvolume ELISA platforms. Passive adsorption was effective for antibody immobilization, while the amino-reactive MCP-2 copolymer ensured stable and functional covalent attachment of protein antigens. DNA-directed immobilization (DDI) demonstrated high versatility. It enables the immobilization of a diverse range of receptors using the Copoly Azide copolymer, which facilitates DBCO-modified DNA attachment for subsequent protein conjugate binding. This strategy allowed the reliable immobilization of complex proteins, expanding the range of usable receptors on a platform. The copolymers also exhibited antifouling properties, minimizing non-specific binding and enhancing assay sensitivity. Designed serological platforms effectively distinguished between COVID-19-positive and -negative serum samples. Quantitative measurements and differentiation between antibody subclasses were also possible. Flexible PET foils increased platform versatility and enable further integration into complex systems, such as microfluidic devices for sophisticated miniaturized diagnostics. Combining DDI with flexible substrates broadens the scope of applications. It facilitates the development of compact, multifunctional platforms suitable for multiplex biosensing and point-of-care diagnostics. The impact of the current study could be broadened by further investigation of the platform’s potential for detecting a wider range of biomarkers, making it applicable to other infectious diseases. The usage of the DDI strategy for different antigens would result in the improvement of the platform’s versatility and adaptability, supporting high-throughput analytical applications. Further development could also focus on enhancing the integration of this platform into portable and field-deployable diagnostic tools, enabling rapid, on-site testing in diverse scenarios.

## Figures and Tables

**Table 1:** Main diagnostic parameters of the assay for the detection of anti-NP IgA and IgG. Results were obtained on the basis of 28 actual samples (sera), including 14 positive and 14 negative samples. MedCalc^®^ Statistical Software version 23.0.2 (MedCalc, Ostend, Belgium) was used for the calculation.

Metric	Value
Sensitivity	100.0%
Specificity	92.9%
Positive Predictive Value (PPV)	93.3%
Negative Predictive Value (NPV)	100.0%
Accuracy	96.4%

## Data Availability

Data are contained within the article and Supplementary Materials.

## Supplementary Materials

The following supporting information can be downloaded at: https://www.mdpi.com/article/10.3390/s24237766/s1, Supplementary Experimental (Reagents, Materials, and Methods), Figure S1: Copolymers composition. A—Chemical structure of MCP-2 and Copoly Azide. B—Monomer molar ratios for MCP-2 and Copoly Azide; Figure S2: Results of SARS CoV-2 antigen selection studies; Figure S3: Results of specificity studies for DDI-type immobilization. Reference [66] is cited in the Supplementary Materials.
