# Supplementary material for: PET Foils Functionalized with Reactive Copolymers as Adaptable Microvolume ELISA Spot Array Platforms for Multiplex Serological Analysis of SARS-CoV-2 Infections
Source: Sensors (Basel). 2024 Dec 4;24(23):7766. doi: 10.3390/s24237766 (PMC11644834; doi:10.3390/s24237766)
Supplement: Supplementary file 1 [file sensors-24-07766-s001.zip › sensors-3293989-supplementary.pdf]

# Supplementary Material

**PET Foils Functionalized with Reactive Copolymers as Adaptable Microvolume ELISA Spot Array Platforms for Multiplex Serological Analysis of SARS-CoV-2 Infections**

## Experimental

### 1. Reagents and Materials

Sodium chloride, potassium chloride, sodium hydrogen phosphate, sodium dihydrogen phosphate, magnesium nitrate, ammonium sulfate, and potassium dihydrogen phosphate were obtained from Sigma-Merck (Poznań, Poland). Ammonia (25%), hydrogen peroxide (30%), sulfuric acid (96%), and sodium hydroxide were obtained from Chempur (Piekary Śląskie, Poland). *p*-nitrophenyl phosphate (PNNP) was obtained from Glentham Life Sciences (Corsham, UK). Anti-human IgG clone line 7701, anti-human IgA clone line 8203 and monoclonal mouse anti-nucleoprotein antibody clone line 1143 were obtained from Medix Biochemica (Espoo, Finland). SARS-CoV Nucleocapsid recombinant protein expressed in *Escherichia coli*, goat anti-human IgG antibody conjugated with alkaline phosphatase, mouse anti-human IgG1, IgG2, IgG3, and IgG4 Fc secondary antibodies, and streptavidin were obtained from Thermo Fisher Scientific (Warsaw, Poland). Rabbit anti-mouse IgG antibody, and goat anti-mouse IgG antibody conjugated with alkaline phosphatase were obtained from Sigma-Aldrich (St. Louis, MO, USA). The proFIRE® Amine Coupling Kit for proteins (>5 kDa) was obtained from Dynamic Biosensors GmbH (Munich, Germany).

**DNA oligonucleotide sequences**

-$NH_2$ ligand strand (48 nt.) 5′-$NH_2$-$C_6$-ATC AGT ACT TGT CAA CAC GAG CAG CCC GTA TAT TCT CCT ACA GCA CTA-3′

- DBCO DNA probe (15 nt.) 5′-DBCO-TEG-TAG TGC TGT AGG AGA-3′

- $NH_2$ DNA probe (15 nt.) 5′-$NH_2$-TAG TGC TGT AGG AGA-3′

- biotin–DNA probe (15 nt.) 5′-biotin-(TEG)-TAG TGC TGT AGG AGA-3′

- ligand strand (48 nt.) 5′ -ATC AGT ACT TGT CAA CAC GAG CAG CCC GTA TAT TCT CCT ACA GCA CTA-3′

## 2. Methods

### 2.1 Conjugation of Receptor Proteins with ssDNA Anchors and Conjugates Purification

The conjugation process was performed using a DNA oligonucleotide with an amine-terminated ligand (3 nmol) and 200 μg of SARS-CoV-2 nucleoprotein or mouse monoclonal antibody against human IgG using the proFIRE® Amine Coupling Kit according to a previously developed protocol [36].

### 2.2 Protein immobilization on polystyrene plates for classic ELISA

To perform standard ELISA, antigens were immobilized onto 96-well polystyrene plates (either non-treated or MediSorp® plates) by adding 50 μL of a 10 μg/mL antigen solution prepared in 50 mM carbonate buffer (pH 9.6) to each well. Wells containing only carbonate buffer were used as references. The plates were incubated overnight at 4°C to allow protein adsorption. After incubation, plates were washed three times with PBST. To block nonspecific binding sites, 50 μL of 3% BSA in PBST was added to each well and incubated for 30 minutes at room temperature. All subsequent antibody or serum dilutions were prepared in PBST containing 3% BSA, and 50 μL of the appropriate solution was incubated in each well for 1 hour at room temperature (antibody concentration ~1 μg/mL unless otherwise specified). Actual sera samples after thawing were diluted 200 times (unless otherwise stated). Each incubation step was followed by three washes with PBST. Subsequently, 50 μL of an antibody-alkaline phosphatase conjugate, diluted to 3 μg/mL in PBST with 3% BSA, was added and

incubated for 1 hour at room temperature. The plate was then washed four times with PBST to remove unbound conjugate. Then, 10 mM freshly prepared PNNP in 50 mM carbonate buffer (pH 9.6) was used as the ALP substrate. The reaction was initiated by dispensing 90 µL of PNNP into each microwell, and detection was performed immediately after substrate addition. Absorbance was measured at 405 nm using a Multiskan Go microplate reader (Thermo Scientific, USA).

**2.3 SPR slides preparation**

SPR gold-coated slides were cleaned under UV/ozone (Ossila Ltd., Sheffield, UK) for 30 minutes, rinsed with deionized (DI) water, and then immersed in a “basic piranha” solution (25% ammonia, 30% hydrogen peroxide, and DI water in a 1:1:3 volumetric ratio) at 70°C for 15 minutes. After rinsing with DI water and drying under compressed air, the slides were soaked in an “acidic piranha” solution (3:1 v/v mixture of concentrated sulfuric acid and perhydrol) for 1 minute. The slides were then rinsed again with DI water and dried.

**2.4 SPR slide modification**

Bare gold-coated slides were treated with a UV/ozone cleaner (Ossila, UK) for 10 minutes and then dip-coated overnight at room temperature with an aqueous solution of Copoly Azide, as described previously. After coating, the slides were rinsed with DI water, dried under compressed air, and cured under vacuum at 80°C for 15 minutes. The Copoly Azide-coated slides were then manually covered with 100 µL of a 0.5 µM DBCO-modified DNA solution and incubated overnight at 4°C in a humid atmosphere. Slides were then rinsed with DI water, dried under compressed air, and prepared for SPR measurements.

For label-free studies and DDI immunoassay design, SPR transducers were covalently modified with streptavidin and coated with a biotin–DNA probe. Streptavidin was immobilized on the PEG-COOH-coated slide by injecting a 50 µg/mL solution in 5 mM acetate buffer (pH 5.0) for 20 minutes, following a previously described protocol [37]. Subsequently, 1 µM

biotin–DNA probe solution in PBST was injected for 20 minutes to immobilize ssDNA, followed by washing with PBST until a stable signal was achieved.

## 2.5 Immobilization of Protein Conjugates and Surface Plasmon Resonance Imaging Measurements

Kinetic studies on the immobilization of ligand strand and protein conjugates were carried out with SPRi Lab Plus instrument (Horiba, France). PBS buffer with 0.05% Tween® 20 (PBST) was used as the running buffer at a flow rate of 80 μL/min. The ligand strand (2.7 μM in PBST) or protein conjugate (~20 nM in 50 mM phosphate buffer, pH 7.2, with 150 mM NaCl) was injected manually (flow rate: 80 μL/min, ~5 min) to monitor DNA-directed protein immobilization in real-time. For regeneration between injections, 50 mM sodium hydroxide was applied at 80 μL/min for 4 minutes. Immunolabeling was performed at the end of each experiment by injecting a 5-minute solution of a specific antibody (anti-mouse IgG or anti-SARS-CoV-2 NP antibodies, 50 μg/mL in PBST).

**Figure S1**

A

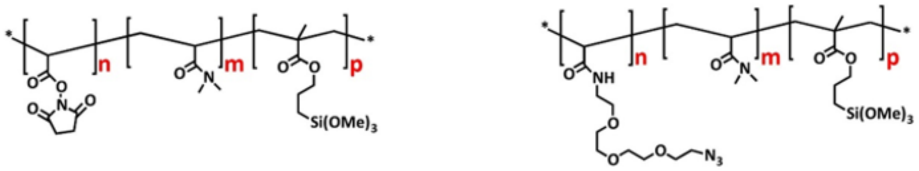

MCP-2 | Copoly Azide

B

| Copolymer | Monomer molar ratio | | |
|---|---|---|---|
| | DMA (m) | NAS/$N_3$ (n) | MAPS (p) |
| MCP-2 | 97 | 2 | 1 |
| Copoly Azide | 95 | 4 | 1 |

C

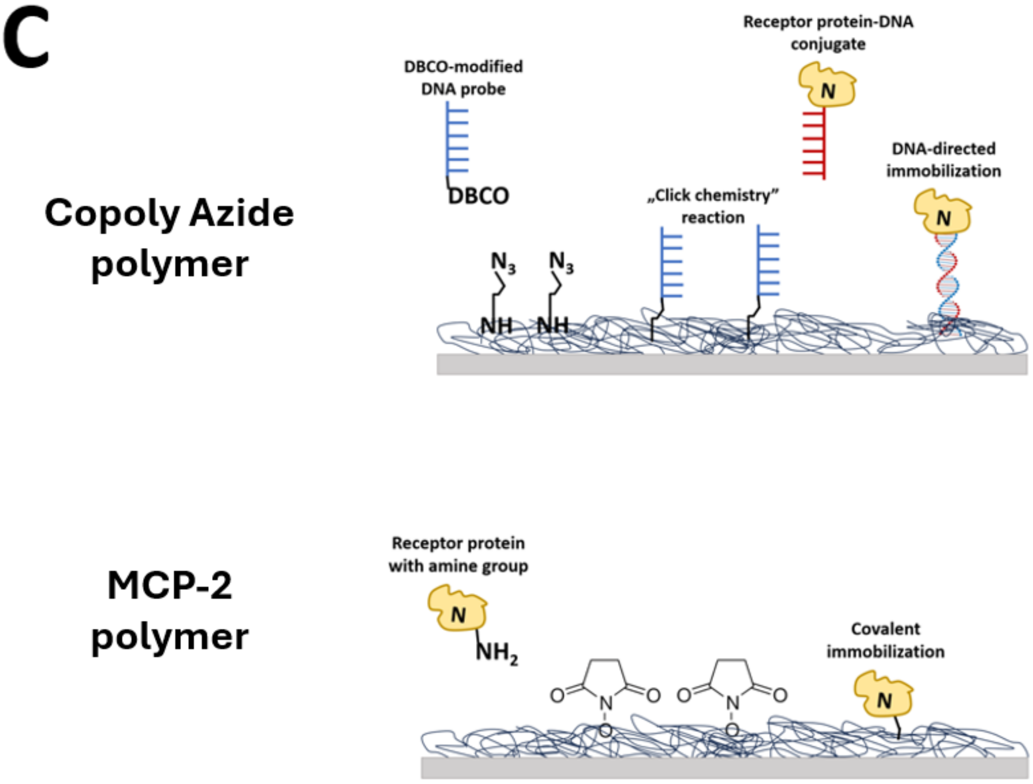


**Fig. S1.** Copolymers composition. A – Chemical structure of MCP-2 and Copoly Azide. B – Monomer molar ratios for MCP-2 and Copoly Azide. C – Schematic mechanism of receptor protein immobilization on PET foil coated with Copoly Azide and MCP-2.

**Figure S2**

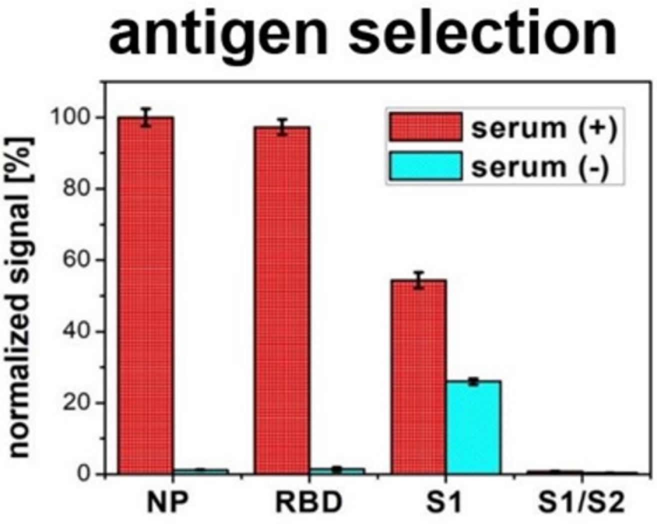


**Fig. S2.** Specific (red) and non-specific (light blue) signals for various SARS-CoV-2 protein receptors.

**Figure S3**

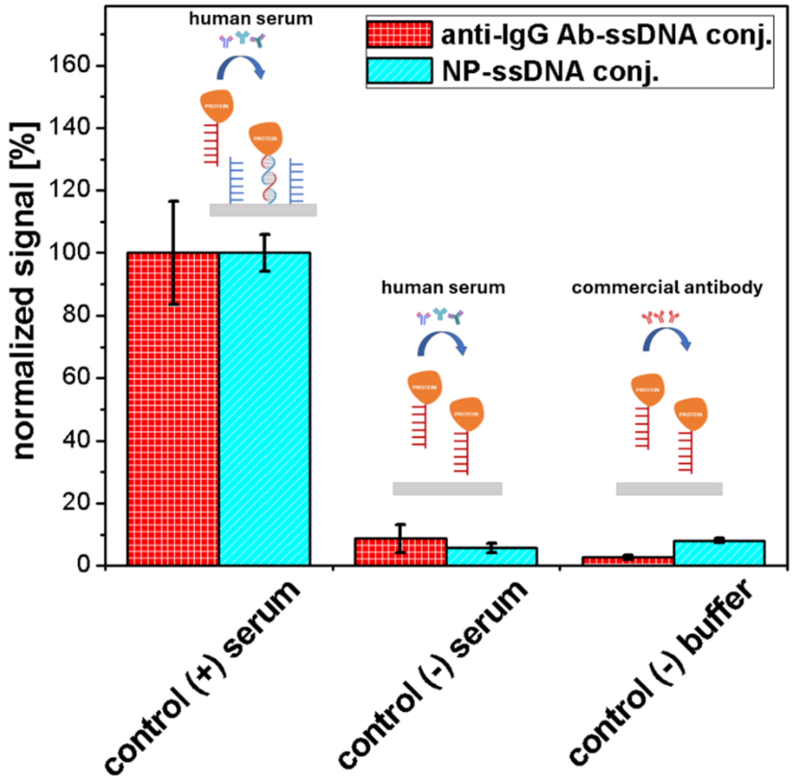


**Fig. S3.** Results of specificity studies for DDI-type immobilization.

## References

[59] **Karoń, S., Drozd, M., & Malinowska, E.** (2024). A Careful Insight into DDI-Type Receptor Layers on the Way to Improvement of Click-Biology-Based Immunosensors. Biosensors, 14(3), 136. https://doi.org/10.3390/bios14030136

[66] **Karoń, S., Porycka, K., Lapitan, L. D. S. Jr., Drozd, M., Pietrzak, M., & Malinowska, E.** (2024). A Versatile Approach to Quality Control of Protein-Based Receptor Layers by Reversible, Nonspecific Staining for Multiplex SPRi Immunosensing. Sensors and Actuators B: Chemical, 136512. https://doi.org/10.1016/j.snb.2024.136512
